# Hazards of the Cytokine Storm and Cytokine-Targeted Therapy in Patients With COVID-19: Review

**DOI:** 10.2196/20193

**Published:** 2020-08-13

**Authors:** Miguel Quirch, Jeannie Lee, Shabnam Rehman

**Affiliations:** 1 Texas Tech University Health Sciences Center Lubbock, TX United States

**Keywords:** coronavirus, COVID-19, convalescent plasma therapy, cytokine storm, SARS-CoV-2, cytokine, immunology, review, mortality, inflammation, therapy

## Abstract

**Background:**

Severe acute respiratory syndrome coronavirus 2 (SARS-CoV-2) has challenged medicine and health care on a global scale. Its impact and frightening mortality rate are in large part attributable to the fact that there is a lack of available treatments. It has been shown that in patients who are severely ill, SARS-CoV-2 can lead to an inflammatory response known as cytokine storm, which involves activation and release of inflammatory cytokines in a positive feedback loop of pathogen-triggered inflammation. Currently, cytokine storm is one of the leading causes of morbidity and mortality in SARS-CoV-2, but there is no proven treatment to combat this systemic response.

**Objective:**

The aim of this paper is to study the cytokine storm response in SARS-CoV-2 and to explore the early treatment options for patients who are critically ill with the coronavirus disease (COVID-19) in the early stages of the pandemic by reviewing the literature.

**Methods:**

A literature review was performed from December 1, 2000, to April 4, 2020, to explore and compare therapies that target cytokine storm among SARS-CoV-2 and prior coronavirus cases.

**Results:**

A total of 38 eligible studies including 24 systematic reviews, 5 meta-analyses, 5 experimental model studies, 7 cohort studies, and 4 case reports matched the criteria.

**Conclusions:**

The severity of the cytokine storm, measured by elevated levels of interleukin-1B, interferon-γ, interferon-inducible protein 10, and monocyte chemoattractant protein 1, was associated with COVID-19 disease severity. Many treatment options with different targets have been proposed during the early stages of the COVID-19 pandemic, ranging from targeting the virus itself to managing the systemic inflammation caused by the virus and the excessive cytokine response. Among the different agents to manage cytokine storm in patients with COVID-19, there is developing support for convalescent plasma therapy particularly for patients who are critically ill or mechanically ventilated and resistant to antivirals and supportive care. Treatment options that were proposed in the beginning phases of the pandemic were multidimensional, and further research is needed to develop a more established treatment guideline.

## Introduction

In December 2019, a novel coronavirus emerged from Wuhan, China. Since then, it has rapidly spread around the world. The coronavirus disease (COVID-19) ranges from a self-limiting upper respiratory tract illness to severe pneumonia, multiorgan failure, and death. It was declared a pandemic by the World Health Organization (WHO) on March 11, 2020 [1-3]. Despite its impact, there is still much uncertainty regarding the frontline treatment of choice for severe acute respiratory syndrome coronavirus 2 (SARS-CoV-2).

Current research indicates that cytokine storm is one of the leading causes of morbidity and mortality in patients with COVID-19. This evidence is based on elevated levels of interleukins (ILs) and other cytokine signaling pathways that are implicated in inflammatory cascades. Zhang et al [4] explored risk factors among patients with COVID-19 who are at higher risk of adverse events such as admission to an intensive care unit (ICU), mechanical ventilation dependence, or death among three hospitals in Wuhan, China from January 13, 2020, to February 26, 2020. As part of their analysis, 28 patients with cancer and COVID-19 were included. Lung cancer was the most frequent type of cancer involved with 7 of 28 (25%) patients; 8 of 28 (29%) patients were suspected to have received COVID-19 from hospital-associated transmission; 15 of 28 (54%) patients had a severe event, with an overall mortality rate of 29% [4]. Receiving antineoplastic therapy within 2 weeks of analysis significantly increased the risk of developing severe events, with a hazard ratio of 4.079 (*P*=.03) [4].

Convalescent plasma therapy is one of the proposed treatments for patients who are critically ill with COVID-19 and have exaggerated inflammatory response to the virus. Plasma therapy has been used in prior viral pandemics such as pandemic influenza A (H1N1), severe acute respiratory syndrome (SARS), Middle East respiratory syndrome (MERS), and Ebola with moderate success. Here, we present a literature review regarding current and potential pharmacologic treatments that specifically aim to target the cytokine storm in the battle against COVID-19.

## Methods

We searched the PubMed and EMBASE databases. We specifically screened studies that were published between December 1, 2000, and April 4, 2020. The following research terms were used: coronavirus, COVID-19, SARS, SARS-CoV-2, cytokine storm, inflammatory response, cytokines, ILs, immunomodulatory, anti-inflammation treatment, and immunocompromised. We excluded studies without detailed methodological reporting. The search was limited to studies published in English. The PRISMA (Preferred Reporting Items for Systematic Reviews and Meta-Analyses) criteria were applied.

The screening of titles and abstracts was performed by the authors. The full texts were reviewed in a second screening. Papers were considered if the study design was a case report, cohort study, series of cases, ecological study, systematic review, meta-analysis, or clinical trial related to the cytokine storm effect and treatment of coronavirus infection.

## Results

**Figure 1:**
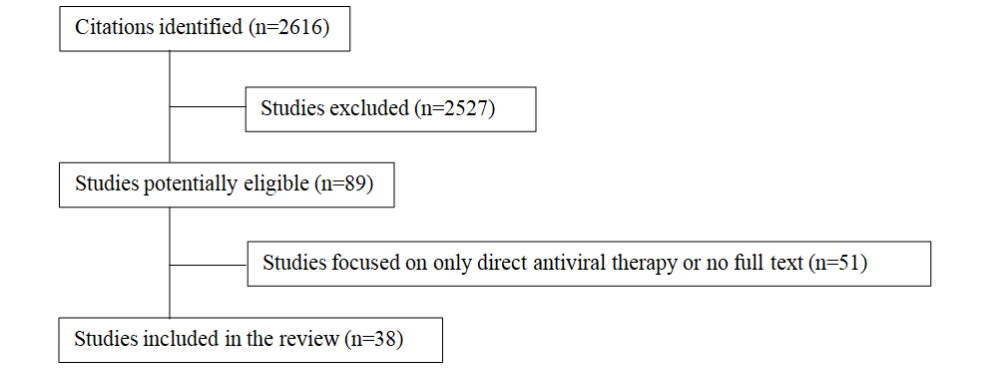
Flow diagram of search results.

Our literature search identified 2616 abstracts, from which 89 potential studies were selected for detailed full-text review. After excluding the studies that were focused on direct antiviral therapy, 41 studies remained. The studies focused on the management of coronavirus infection and cytokine storm. No nonpeer reviewed publications were excluded from the study. Studies that were not available in full text were excluded. A total of 38 studies were included in the final analysis. Of these, 24 systematic reviews, 5 meta-analyses, 5 experimental model studies, 7 cohort studies, and 4 case reports were selected. Some studies contributed to more than one section in this review (Figure 1).

## Discussion

### Principal Findings

The pathogenesis of cytokine storm in patients who are critically ill with COVID-19 involves a dysregulation of the immune response. Excessive release of cytokines causes damage on a local and systemic level. Elevated cytokine responses have been associated with a high rate of morbidity and mortality. Our review explored multiple treatment strategies, ranging from direct antiviral therapy to glucocorticoids to those that specifically target the cytokine storm effect, such as immunomodulators and plasmapheresis (Table 1). As there are currently no well-established treatment guidelines, research in targeting the cytokine storm is crucial in management at the earliest stage possible.

**Table 1:** Clinical management studies in viral outbreaks.

Treatment	SARS-CoV-2^a^	MERS^b^	H1N1^c^	SARS^d^
Glucocorticoids	Wang et al^e^ [5] WHO^f^ [6]	Russell et al^g^ [7]	WHO [6]	Russell et al^g^ [7]
IL^h^-6 antibody	Xu et al^i^ [8] Sanofi [9] Gritti^i^ [10]	N/A^j^	N/A	N/A
IL-1 antibody	Nicastri^i^ [11] Sobi [12]	N/A	N/A	N/A
CQ^k^/HCQ^l^	Zhang et al^i^ [13] Dong et al^g^ [14] Gautret et al^i^ [15]	N/A	N/A	Zhang et al^g^ [16]
JAK2^m^ inhibitors	Wu and Yang^i^ [17]	Wu and Yang^i^ [17]	Wu and Yang^i^ [17]	N/A
Antioxidants	Zhang et al^g^ [18] Peng^i^ [19]	Zhang et al^g^ [18]	N/A	Zhang et al^g^ [18]
Plasma therapy	Tanne [20] Roback and Guarner [21] Shen et al^i^ [22]	Chun et al^e^ [23]	Hung et al^i^ [24]	Cheng et al^i^ [25]

^a^SARS-CoV-2: severe acute respiratory syndrome coronavirus 2.

^b^MERS: Middle East respiratory syndrome.

^c^H1N1: pandemic influenza A.

^d^SARS: severe acute respiratory syndrome.

^e^Case report.

^f^WHO: World Health Organization.

^g^Review.

^h^IL: interleukin.

^i^Series of cases/observational studies.

^j^N/A: not applicable.

^k^CQ: chloroquine.

^l^HCQ: hydroxychloroquine.

^m^JAK2: Janus kinase 2.

### Cytokine Storm and the Impaired Immune System

The coronavirus pneumonia, such as SARS and MERS, has demonstrated that a combination of severe inflammation, oxidative stress, and an exaggerated immune response contributes to the COVID-19 pathology. This uncontrolled and excessive load of proinflammatory cytokines, referred to as a cytokine storm, can lead to acute lung injury, acute respiratory distress syndrome (ARDS), and even death. Huang et al [26] reported that patients who are critically ill with COVID-19 have an elevated cytokine profile that resembles that of cytokine storms reported in SARS and MERS. The prospective cohort study from Wuhan, China measured the cytokine levels in 41 patients. Out of 41 patients, 13 (32%) were admitted to the ICU for high-flow nasal cannula or higher-level oxygen support requirements. The majority of the patients in this cohort presented with fever, dry cough, and dyspnea. Chest computed tomography (CT) scans showed bilateral ground-glass opacities, and some patients even progressed to ARDS in just 2 days.

The pathophysiology of COVID-19 patients with respiratory compromise is attributed to the diffuse alveolar damage that SARS-COV-2 causes, similar to what was reported in prior patients with SARS. Although the viral infection targets the alveolar epithelial cells, the coronavirus is known to have a long incubation period. During this time, the virus can cause multi-organ damage, specifically affecting the spleen, lymph nodes, small blood vessels, heart, liver, and kidney. Consequently, the impaired organs that make up the immune system lead to lymphocytopenia, which interferes with clearance of the virus. Autopsies from patients with COVID-19 and pathology from those who had pneumonectomy for lung cancer have shown varying degrees of diffuse alveolar damage, including type II epithelial cell hyperplasia, alveolar cavity fibrosis, and microvascular thrombus [27].

In addition to the virus itself causing alveolar damage, an immunocompromised system poses further challenge to treating patients who are critically ill with COVID-19 that have elevated levels of IL-1β, interferon (IFN)–γ, IFN-inducible protein 10 (IP-10), and monocyte chemoattractant protein 1 (MCP-1), which activate T-helper (Th)1 cell responses. Patients who required ICU admission had higher concentrations of granulocyte colony-stimulating factor, IP-10, MCP-1, macrophage inflammatory protein 1A, and tumor necrosis factor alpha (TNF-α) than did those not requiring ICU admission. Overall, Huang et al [26] suggested that the cytokine storm was associated with disease severity. Despite supportive treatment, mortality was as high as 6 out of the 41 (15%) patients in this cohort [26].

Early studies on patients with SARS showed that elevation of proinflammatory cytokines such as IL-1β, IL-6, IL-12, IFN-γ, IP-10, and MCP-1 was associated with pulmonary inflammation and extensive lung damage [26]. A prospective cohort study by Wong et al [28] found that patients with SARS exhibited a significant increase in the antiviral cytokine IFN-γ and proinflammatory cytokines (IL-1β, IL-6 and IL-12) during a cytokine storm, with a moderate increase in anti-inflammatory IL-10 in only some patients. This selective IL-10 response was not further analyzed. The early elevation of inflammatory cytokines was attributed to the SARS-induced activation of Th1 cells and natural killer (NK) cells, resulting in pulmonary inflammation. In a former clinical study by Wong et al [29], 128 of 157 (82%) patients with SARS developed neutrophilia associated with ICU admission and mortality from pneumonia [29]. Therefore, studies indicate that polymorphonuclear leukocyte–induced acute pneumonitis in the early stages of SARS-CoV-2 infection contributes to the later development of ARDS and ultimately the pulmonary destruction in patients with SARS.

A similar elevation of inflammatory cytokines has been reported among patients with MERS, although the theory of cytokine storm has been much less studied during the MERS outbreak. A study by Mahallawi et al [30] compared the profile of cytokine responses in plasma samples from patients with MERS (n=7) versus those of the healthy controls (n=13). A strong proinflammatory Th1 and Th17 response was seen in patients with MERS, with markedly increased concentrations of proinflammatory cytokines (IFN-γ, TNF-α, IL-15, and IL-17), that elicited a type II IFN response with innate and acquired immunity interfering with viral replication [27,31]. Patients with MERS also had a marked increase in IFN-α2 concentration compared to the healthy controls (*P*<.01). Patients who were infected had a significant increase in IFN-α2 ranging from 26- to 71-fold increase when compared to healthy controls, indicating a type I IFN response as first line defense against the viral infection [27].

### Lymphocytopenia and Inflammatory Response

Lymphocytopenia is a prominent marker of COVID-19 that is a diagnostic criteria for COVID-19 in China. Both T cell and NK cell counts are depressed in patients with COVID-19, with pronounced reduction noted among the more severe cases [32]. A study by Zhang et al [16] examined patients with COVID-19 in Wuhan, China. Patients were categorized as having mild versus severe infection. Patients who were severe were those with extrapulmonary systemic hyperinflammation syndrome and required ICU care [16]. Among these patients, CD8+ T cells were reported to be reduced by 28.43% and 61.9% in mild and severe groups, respectively, while NK cells were reduced by 34.31% and 47.62% in mild and severe groups, respectively [16]. According to prior studies, the more severe cases of COVID-19 were associated with acute lymphocytopenia with destruction of lymphoid tissues including the spleen and lymph node. Immunohistochemical staining showed that CD4+ T cells and CD8+ T cells were decreased, which was associated with an overexpression of proinflammatory cytokines and chemokines. Although the mechanism is not conclusive, studies ultimately attribute lymphocytopenia in patients who were critically ill with COVID-19 to a dysregulation of the immune system [33].

The overall mechanism that leads to rapid respiratory failure in the setting of excessive inflammatory response remains unclear. Prominent theories propose a direct invasion via angiotensin converting enzyme 2 (ACE 2) versus a systemic inflammatory response via the cytokine storm effect. Given that there have been no findings of ACE 2 expression on lymphocytes, it is largely theorized that the lymphocyte destruction is due to cytokine storm alone. A study by Zhang et al [16] showed that in type II alveolar epithelia and macrophages, virus inclusion bodies were detected, indicating a primary cytokine storm effect. Regardless of the underlying mechanism, anti-inflammatory treatment specifically targeting the cytokine storm effect seems vital to the survival of patients who are critically ill with COVID-19, though there is a lack of evidence at this time supporting effective treatments.

### Glucocorticoid Therapy

The effect of glucocorticoids has been extensively studied with SARS and MERS in the past. During the SARS outbreak in 2003, it became the primary immunomodulatory therapy with mixed results. Although glucocorticoids improved fever and oxygenation in many cases, other studies showed adverse reactions, including delayed virus clearance or even further worsening of the disease. Currently, glucocorticoids are used to suppress cytokine storm symptoms and improve ARDS.

A retrospective review of 138 patients in Wuhan, China by Wang et al [5] examined characteristics of hospital-related transmission of the illness. The review also analyzed a variety of treatments including antivirals, antibiotics, and glucocorticoids. Although it did not control for any variables, the study found significant treatment differences between patients in the ICU and those not in the ICU, with a trend toward patients in the ICU receiving glucocorticoids. Out of 138 patients, 62 (44.9%) received glucocorticoids. Of the 36 patients admitted to the hospital and transferred to the ICU, 26 (72%) patients were receiving glucocorticoids.

Russel et al [7] reported in a literature review that patients during the SARS and MERS outbreaks who received glucocorticoids were more likely to require mechanical ventilation, vasopressor support, and renal replacement therapy. It also did not improve 90-day mortality (adjusted odds ratio 0.8, 95% CI 0.5-1.1; *P*=.12). Rather, the results indicated delayed clearance of viral RNA from respiratory tract secretions (adjusted hazard ratio 0.4, 95% CI 0.2-0.7; *P*<.001) [7]. Because of the overall poor clinical support for glucocorticoid therapy, the current WHO guideline does not support glucocorticoid use for treatment of viral pneumonia and ARDS for COVID-19 cases without concurrent conditions such as chronic obstructive pulmonary disease or asthma that would independently require glucocorticoid use [6].

### IL-6 Antibodies

#### Tocilizumab

Tocilizumab (TCZ) is a recombinant human IL-6 monoclonal antibody that inhibits IL-6 signaling and mediation of inflammatory response. Xu et al [8] conducted a retrospective study to determine the efficacy of TCZ in addition to the typical treatment with Lopinavir, Methylprednisolone, and supplemental oxygen to treat patients who are severely ill with COVID-19 [8]. Severity was defined by the Diagnosis and Treatment Protocol for Novel Coronavirus Criteria sponsored by the National Health Commission of the People’s Republic of China and was defined if one of the following conditions were met: respiratory rate≥30 breaths/minute, SpO2≤93% while breathing room air, or PaO2/FiO2 ratio≤300 mmHg. TCZ 400 mg was intravenously administered once to 21 patients. Prior to treatment with TCZ, inflammatory markers including c-reactive protein (CRP) and IL-6 were measured, with a mean CRP value of 75.06 mg/L and mean IL-6 level of 132.38 pg/ml [8]. Patients’ fevers improved within a few days, and 15 of 20 (75%) patients that required oxygen had improved oxygenation, with opacity of lung lesions on CT scans showing improvement in 19 of 21 (91%) patients. The percentage of peripheral lymphocytes returned to normal in 10 of 19 (53%) patients with measured levels. Additionally, CRP levels returned to normal in 16 of 19 (84%) patients by the fifth day of treatment. IL-6 levels, unfortunately, were not measured after treatment [8]. Overall, the data suggested that TCZ may be an effective treatment in severe COVID-19 cases. Based on promising results from the Xu et al [8] study, Sanofi and Regeneron pharmaceuticals received approval to open a clinical trial on Sarilumab, another anti–IL-6 monoclonal antibody in mid-March 2020 [9].

#### Siltuximab

Siltuximab is an IL-6 targeted monoclonal antibody that has been approved by numerous regulatory bodies including the Food and Drug Administration (FDA) in the treatment of Castleman’s disease who are negative for HIV and human herpesvirus-8. Gritti et al [10] began enrollment into the Siltuximab in Serious COVID-19 study at the Papa Giovanni XXIII Hospital in Bergamo, Italy in March of 2020 as a retrospective cohort study examining patients receiving siltuximab versus matched controls [10]. Their primary endpoints were the need for invasive ventilation, time spent in ICU, and 30-day mortality. Preliminary results included 21 patients that received a dose of 11 mg/kg siltuximab given over 1 hour with a second dose per investigator discretion. There were 5 patients that received the second dose 48-72 hours after the initial infusion. Clinical improvement was observed in 7 of the 21 (33%) patients, 9 (43%) patients stabilized clinically (no change or worsening in condition), and 5 (24%) patients experienced worsening in condition. Of the 5 that worsened, 1 died and 1 experienced a stroke. CRP levels were measured in 16 patients, with improvement in the days after administration. The study is currently ongoing, and the currently released data is promising in that it offers a potential therapy for COVID-19.

#### Sarilumab

Sarilumab is an additional IL-6 targeted monoclonal antibody that has been approved in the treatment of rheumatoid arthritis [9]. TThere is currently no data for the use of sarilumab in viral syndromes. Given available data for TCZ, additional IL-6 therapies are being investigated. There is currently a phase II/III randomized, double-blind, placebo-controlled trial to evaluate response in patients who are severely ill with COVID-19. The primary endpoint is reduction of fever, and the secondary endpoint is decreased demand for supplemental oxygen.

### IL-1 Antibodies

Anakinra is a targeted monoclonal antibody IL-1 receptor antagonist that is being investigated. Due to promising current data on TCZ, anakinra is being investigated in a phase II/III open label, controlled, parallel, three arm study versus Emapalumab or standard of care [11]. Emapalumab is a monoclonal antibody against IFN-γ, which is currently being used in the treatment of hemophagocytic lymphohistiocytosis, a life-threatening disease caused by excessive immune activation [12].

### Chloroquine and Hydroxychloroquine

Chloroquine (CQ) and hydroxychloroquine (HCQ) have been used for decades as the primary choice for prophylaxis and treatment of malaria but also have had effect on the Marburg virus, Zika virus, dengue virus, Ebola virus, and SARS. CQ blocks viral infection by altering the endosomal pH that is required for viral particles to bind to the cell surface receptor. CQ also interferes with the glycosylation of SARS cellular receptors, specifically ACE 2. Its immunomodulatory, anti-inflammatory, and antiviral mechanism works synergistically, making it a choice for their efficacy and side-effect profile [16].

An early clinical trial conducted in patients with COVID-19 in China showed better clinical outcome and earlier viral clearance in patients treated with CQ compared to control groups [13,14]. Based on these positive findings, Chinese experts recommended that patients with mild, moderate, and severe cases of COVID-19 pneumonia be treated with 500 mg CQ twice per day for 10 days [14].

A clinical trial by Gautret et al [15] studied the effect of HCQ in patients with SARS-CoV-2 from hospitals in South France. A total of 26 patients received oral HCQ 200 mg three times per day for 10 days, and 16 were control patients. Among the HCQ-treated patients, 6 patients also received azithromycin (500 mg on day 1, followed by 250 mg per day for the next four days) to prevent bacterial superinfection. Each day, patients received a standardized clinical examination along with a nasopharyngeal sample collection. Additionally, 6 patients treated with HCQ were lost in follow-up by survey due to early cessation of treatment. Despite a small sample size, the study showed that the HCQ treatment was associated with clinical improvement, faster viral load reduction, and effect reinforcement with azithromycin [13]. At day 6 postinclusion, 14 of the 20 (70%) HCQ-treated patients were virologically cured compared with the 2 of 16 (13%) in the control group (*P*=.001). All patients treated with HCQ and azithromycin combination were virologically cured compared to the 8 of 14 (57.1%) in patients treated with HCQ and 2 of 16 (13%) in the control group (*P*<.001). One patient who was still polymerase chain reaction (PCR)–positive on day six postinclusion under HCQ treatment only received azithromycin in addition to the HCQ on day eight postinclusion; the viral load was cleared on day nine postinfection. The clearing of the viral nasopharyngeal carriage of SARS-CoV-2 is critical, as studies have shown that the mean duration of viral shedding in patients with COVID-19 in China was 20 days [13]. Given the prolonged viral shedding period, transmissibility is a critical issue, and further investigation and trials are needed to determine the efficacy of these agents.

### Janus Kinase 2 Inhibitors

Patients with severe COVID-19 syndrome have elevated counts of Th17 cells contributing to the development of cytokine storm. Wu and Yang [17] proposed the use of the Janus kinase 2 (JAK2) inhibitor fedratinib for the suppression of Th17 cytokine production. JAK2 works through signal transducer and activator of transcription (STAT)3 to mediate IL-6 and IL-23 signals for Th17 cells. JAK1 and tyrosine kinase 2 receptors work through STAT1 and STAT2, which are important to the function of antiviral immunity. Wu and Yang [17], thus, proposed selective targeting of JAK2 inhibition to mitigate or even prevent the cytokine storm in COVID-19. In their previous testing in vitro, they found that Fedratinib decreased expression of IL-17 and IL-22 expression [17]. Fedratinib is currently approved by the FDA for use in myeloproliferative neoplasms that take advantage of the JAK2 pathway, such as polycythemia vera; however, it has not been tested in the setting of viral syndromes yet.

### Targeting the Antioxidant Effect

Other therapies such as melatonin and vitamin C have been implicated in anti-inflammatory and antioxidant effects, and could potentially prove beneficial in the COVID-19 setting. There is currently a clinical trial underway for vitamin C; however, melatonin has not been examined yet at this time.

#### Melatonin

Although many therapies have been proposed based on previous use in viral pandemics, others are proposed simply due to beneficial effects seen in other nonviral conditions. Melatonin, a drug typically used for insomnia, has been found to have beneficial effects in other diseases such as atherosclerosis and respiratory distress. Per Zhang et al [18], in contrast to other coronaviruses, SARS-CoV-2 infection leads to increased levels of IL-1β, IFN-γ, IP-10, and MCP-1, in addition to IL-4 and IL-10, which was diminished in many patients with SARS. The study suggested that melatonin mediates anti-inflammatory effects through sirtuin 1, which inhibits proteins, down regulates macrophages toward proinflammation, and attenuates lung injury and inflammation. It has also been shown to suppress proinflammatory cytokines and regulate nucleotide-binding oligomerization domain-like receptors 3 in radiation-induced lung injury.

Melatonin may also have antioxidative effects in the setting of ARDS. Previous studies by Gitto et al [34] used melatonin in newborns with respiratory distress with success due to antioxidant and anti-inflammatory effects. In a study of patients with multiple sclerosis, melatonin has been shown to reduce serum concentrations of TNF-α, IL-6, IL-1β, and lipoperoxides, further leading to evidence that it may reduce inflammatory cytokines [35]. Testing melatonin in ICU patients in the past has not shown significant adverse events. Given the high safety profile, Zhang et al [18] proposed the use of melatonin for study in patients with COVID-19 [36].

#### Vitamin C

Vitamin C, or ascorbic acid, is a water-soluble vitamin that aids in the synthesis of collagen in connective tissue and may also work as an antioxidant. Studies in chicken embryo cultures demonstrated that vitamin C provided resistance to coronavirus infection. Furthermore, there have been human trials that are suggestive of a lower incidence of pneumonia in groups on vitamin C treatment [19,37].

Given previous data on vitamin C, there is currently an ongoing prospective randomized clinical trial in Wuhan, China examining 7 days of high dose intravenous (IV) vitamin C therapy versus placebo with endpoints being ventilator requirements, vasopressor requirements, ICU length of stay, and 28-day mortality. The full results of the study are expected to be released by September 2020 [19].

### Convalescent Plasma Therapy

The search for COVID-19 treatment has extended to plasma therapy, which has been used in previous viral outbreaks such as SARS, Ebola, and H1N1. Convalescent plasma therapy relies on the purified plasma obtained usually by apheresis from patients that have recovered from the particular infection and subsequently transfused to ABO-compatible patients with the active disease. Given previous success, this therapy is currently being proposed in patients who are critically ill who have failed more conservative management. In fact, as of March 2020, the FDA approved the use of convalescent plasma therapy to treat patients who are critically ill with COVID-19, provided that the treating physicians apply via the process for an investigational new drug application [20]. Here, we aim to discuss plasma therapy in patients who are critically ill, as it has been used in prior respiratory viral outbreaks and potential applications in the current pandemic, including efficacy in inflammatory syndromes.

#### SARS Treatment in Hong Kong in 2003

During the height of the SARS epidemic in 2003, Cheng et al [25] explored the efficacy of convalescent plasma therapy. The study included 339 patients that were admitted at the Prince of Wales Hospital in Hong Kong between March 20 and May 26 of 2003 for SARS. Initial treatment was with Cefotaxime and Levofloxacin (or clarithromycin) to treat community-acquired pneumonia. If fever persisted, patients were treated with Ribavirin at 1200 mg per os *ter in die* or IV 400 mg every 8 hours and Prednisolone (0.5-1 mg/kg) on day three. Patients with radiographic progression and hypoxemia were given pulsed methylprednisolone 500 mg IV daily for 2-3 doses. For patients that continued to deteriorate per SaO2<90% on 0.5 FiO2, 200-400 ml (4-5 ml/kg) of ABO-compatible convalescent plasma obtained from recovered patients with SARS was administered. Out of 339 patients, 80 (24%) received convalescent plasma treatment.

Of the 80 patients selected for convalescent plasma treatment, 33 (41%) had good outcomes (discharge by day 22), and 47 (59%) had poor outcomes (hospitalization beyond 22 days or death). The median age of patients with good outcomes was 37.9, and the median age of patients with poor outcomes was 50.2 (*P*<.001). The median day of plasma infusion was 11.7 for patients with good outcomes and 16 for patients with poor outcomes (*P*<.001). Patients given convalescent plasma before day 14 tended toward a better outcome, with a mortality of 6.3% (3/48) versus 22% (7/32) in the after day 14 group (*P*=.08). Out of 33 patients with good outcomes, 20 (61%) were PCR-positive and seronegative for coronavirus compared to 10 of 47 (21%) with poor outcome (*P*<.001). The 30 patients that were PCR-positive and seronegative at the time of convalescent plasma therapy had better outcomes than patients already seropositive, 67% (20/30) versus 20% (10/50) (*P*=.001). There were no reported adverse effects after administration of plasma infusions; however, the primary limitation of plasma convalescent therapy was availability. The mortality rate recorded in the study was 13% (10/80), while the SARS mortality rate in Hong Kong during that same time period was 17% (299/1755) from March 6 to May 24, 2003 [25].

#### H1N1 Treatment in Hong Kong in 2009

During the H1N1 virus pandemic of 2009, Hung et al [24] examined the effects of convalescent plasma treatment in patients who were critically ill in Hong Kong via a prospective cohort study. From September 1, 2009, to June 30, 2010, patients older than 18 years with H1N1 infection requiring intensive care were offered treatment with convalescent plasma harvested via apheresis from suitable donors. A total of 93 patients were recruited with 20 patients receiving plasma therapy. The remaining patients declined plasma treatment and were included as untreated controls. Donors were selected by reverse transcriptase-PCR (RT-PCR)–positive influenza A virus M and pandemic H1 genes and negative RT-PCR testing of seasonal influenza A virus H1 and H3 genes with clinical recovery from infection at least 2 weeks in addition to meeting standard plasma donation criteria. All patients received treatment with oseltamivir and inhaled zanamivir for influenza.

Of the patients that received convalescent plasma therapy versus patients who were untreated, mortality was found to be 20% (4/20) versus 55% (40/73) with *P*=.01 [24]. Overall, the mortality was 47% (44/93). Other measured factors included viral load, IL-6, IL-10, and TNF-α, which were all found to be significantly lower in the treatment group on subsequent measurements. No reported adverse events were recorded from plasma therapy. 

#### SARS-CoV-2 Treatment in Shenzhen, China in 2020

One of the earliest studies of convalescent plasma therapy for SARS-CoV-2 was done by Shen et al [22] in Shenzhen, China. From January 20, 2020, through March 25, 2020, at Shenzhen Third People’s Hospital, 5 patients who were critically ill with COVID-19 were treated with convalescent plasma transfusion [22]. Selection criteria included COVID-19 diagnosis via RT-PCR, severe pneumonia with rapid progression (P_a_O_2_/F_i_O_2_<300 with mechanical ventilatory support), and continuously high viral load despite antiviral treatment.

Treatment prior to plasma therapy included methylprednisolone, lopinavir/ritonavir, IFN-α-1b, arbidol, and favipiravir. One day prior to receiving treatment, the patient’s blood was collected and enzyme-linked immunosorbent assay (ELISA) and neutralizing antibody titers were tested. On the day of treatment, ABO blood type and compatibility with convalescent plasma donors were measured. Patients were then treated with two consecutive transfusions of 200-250 mL of ABO-compatible convalescent plasma for 400 mL in total with ongoing treatment for antiviral agents until SARS-CoV-2 viral loads were negative [22]. 

Convalescent donors were between 18 and 60 years of age, and had recovered from SARS-CoV-2 infection. They were previously diagnosed with COVID-19 by a quantitative RT-PCR and tested negative for SARS-CoV-2, respiratory viruses, hepatitis B, hepatitis C, HIV, and syphilis. They were also required to be asymptomatic for at least 10 days and have SARS-CoV-2 specific ELISA antibody titer higher than 1:1000 and a neutralizing antibody titer greater than 40. Donation of 400mL of convalescent plasma was then obtained by apheresis and immediately transfused to recipients [22].

Administration of plasma was between 10 and 22 days with the median being day 20. Patients were between 30 and 70 years of age. Of the 5 patients, only 1 had pre-existing conditions of hypertension and mitral insufficiency, and none had a history of smoking. The measured characteristics before and after transfusion included body temperature, cycle threshold value, procalcitonin, CRP, and IL-6 levels [22].

The results noted improvement of cycle threshold value 1 day posttransfusion, with negative testing achieved between days 1 and 12 posttransfusion. IL-6, CRP, and procalcitonin trended downward. Of the 5 patients, 3 were able to be weaned from ventilation, and one was able to be weaned from extracorporeal membrane oxygenation to mechanical ventilation. Donor’s plasma receptor binding domain immunoglobulin (Ig)G and IgM, and neutralizing antibody titers were measured via a cell culture to determine inhibition activity against SARS-CoV-2 [22].

#### Risks and Challenges to Plasma Therapy

Convalescent plasma therapy involves strict quality control to ensure that there is adequate neutralizing antibodies present in the obtained plasma. However, even with quality control, it is not without risk. The risks of convalescent plasma include the same risks as those with transfusion of any blood products: anaphylactic shock, transfusion-associated circulatory overload, and transfusion-related acute lung injury (TRALI).

During the MERS outbreak in May 2015, convalescent plasma therapy was used in many patients. Chun et al [23] described 1 such patient that experienced an unfortunate complication from this therapy. The patient was a 32-year-old male who was admitted and diagnosed with MERS and subsequently treated with ribavirin and lopinavir/ritonavir, as well as a single dose of IFN-α-2a. ABO/RhD-compatible convalescent plasma was then obtained from a patient who had previously recovered from MERS. Within 2 hours of transfusion, the patient developed respiratory distress that was concerning for TRALI despite apparent compatibility. Viral load was found to be reduced in the patient, and retrospective analysis was not able to trace anti–human leukocyte antigen (HLA) or anti-HNA antibodies.

Investigation into these issues has shown that the additive risk of HLA in female donors with one childbirth can be as high as 14.5%, arguing for a preference toward male donors to reduce this risk [38-40]. Since then, many studies and authors have suggested the use of only male donors citing a previous review [39].

In addition, the limitations in the case series published by Shen et al [22] and Roback and Guarner [21] in their editorial to JAMA reported difficulties in finding standard antibody doses from donors of convalescent plasma [21,22]. Therefore, they proposed combining convalescent plasma with hyperimmune globulin (H-Ig). Unlike convalescent plasma, H-Ig has standardized antibody doses from collecting plasma samples from patients with high antibody titers and filtering out IgM antibodies. H-Ig and convalescent plasma may both be stored for years. Monitoring patient response in comparison to objective lab values such as antibody titers and neutralizing activity could help identify which patients can be donors or recipients [21].

### Conclusion

SARS-CoV-2 is a global pandemic that has challenged the world of medicine, research, and health care. The purpose of this paper is to highlight the important current and developing therapies in the management of severe COVID-19, in particular, therapies that can aid in the syndrome known as cytokine storm.

Immunomodulators such as siltuximab, anakinra, sarilumab, fedratinib, and TCZ target different components of the inflammatory cascade involved in cytokine storm and are in clinical trials. Data is currently strongest for TCZ; however, the results of these studies are not all ready, and investigators may wish to evaluate if patients could be candidates for any one of these trials. 

Although convalescent plasma therapy works by using passively produced antibodies from patients recovered from the illness to treat patients in active disease states, a preliminary case series from China has found that there may also be activity that reduces the markers of cytokine storm such as IL-6. Further use of this therapy is underway in multiple nations around the world in various clinical trials, and it is a promising avenue of treatment for patients who are critically ill.

The treatment for COVID-19 has been met with dire need and urgency during this global pandemic. Current treatments are essentially limited to supportive care and treatment of secondary infections without directly targeted therapies to either the virus or the hypothesized cytokine storm. Although there are a number of clinical trials underway with constantly new therapies being proposed for patients who are critically ill, further study and concrete data are needed to establish treatment guidelines.

## Abbreviations

ACE 2: angiotensin converting enzyme 2
ARDS: acute respiratory distress syndrome
COVID-19: coronavirus disease
CQ: chloroquine
CRP: c-reactive protein
CT: computed tomography
ELISA: enzyme-linked immunosorbent assay
FDA: Food and Drug Administration
H1N1: pandemic influenza A
HCQ: hydroxychloroquine
HLA: human leukocyte antigen
H-Ig: hyperimmune globulin
ICU: intensive care unit
IFN: interferon
Ig: immunoglobulin
IL: interleukin
IP-10: interferon-inducible protein 10
IV: intravenous
JAK2: Janus kinase 2
MCP-1: monocyte chemoattractant protein 1
MERS: Middle East respiratory syndrome
NK: natural killer
PCR: polymerase chain reaction
PRISMA: Preferred Reporting Items for Systematic Reviews and Meta-Analyses
RT-PCR: reverse transcriptase-polymerase chain reaction
SARS: severe acute respiratory syndrome
SARS-CoV-2: severe acute respiratory syndrome coronavirus 2
STAT: signal transducer and activator of transcription
TCZ: tocilizumab
Th1: T-helper
TNF-α: tumor necrosis factor alpha
TRALI: transfusion-related acute lung injury
WHO: World Health Organization
